# Outcome preferences in fidelity-adaptation scenarios across evidence-based parenting programs: A discrete choice experiment

**DOI:** 10.1186/s13012-025-01421-y

**Published:** 2025-02-18

**Authors:** Kristoffer Pettersson, Philip Millroth, Fabrizia Giannotta, Pernilla Liedgren, Aaron R. Lyon, Henna Hasson, Ulrica von Thiele Schwarz

**Affiliations:** 1 School of Health, Care and Social Welfare, Mälardalen University, 721 23 Västerås, SE Sweden; 2 Department of Psychology, Uppsala University, 752 37 Uppsala, SE Sweden; 3 Department of Public Health Sciences, Stockholm University, 106 91, Stockholm, SE Sweden; 4 Department of Psychiatry and Behavioral Sciences, University of Washington, Seattle, WA USA; 5 Procome Research Group, Department of Learning, Informatics, Management and Ethics, Medical Management Centre, Karolinska Institutet, 171 77 Stockholm, SE Sweden; 6 Center for Epidemiology and Community Medicine (CES), Stockholm County Council, 171 29 Stockholm, SE Sweden; 7 Faculty of Caring Science, Work Life and Social Welfare, Department of Work Life and Social Welfare, University of Borås, Borås, Sweden; 8 Department of Psychology, University of Turin, via verdi 10, Turin, 10124 Sweden

**Keywords:** Adaptation, Discrete choice experiment, Parenting programs, Outcome preference, Trade-offs, Dilemmas

## Abstract

**Background:**

Implementing evidence-based parenting programs often involves navigating fidelity-adaptation decisions. While research has explored various aspects of this dilemma, little is known about how practitioners’ outcome preferences influence their decisions in real-world scenarios.

**Methods:**

This study employed a discrete choice experiment (DCE) to investigate the relative importance of five outcomes (Relationship Quality, Satisfaction, Workload Strain, Value Conflict, and Reach) in fidelity-adaptation decisions among 209 practitioners delivering evidence-based parenting programs in Sweden. The DCE presented 25 choice sets across five contextual scenarios, analyzed using Bayesian hierarchical logistic regression.

**Results:**

All five outcomes significantly influenced practitioners’ choices, with Relationship Quality emerging as the most impactful (log-odds: 4.56, 95% CI [4.16, 4.91]). Satisfaction and minimizing Value Conflict showed similar importance (log odds: 2.45 and -2.40, respectively), while Workload Strain and Reach had slightly less impact (log odds: -2.10 and 1.96, respectively).

**Conclusions:**

This study offers a novel perspective on the role of outcome preference in navigating fidelity-adaptation decisions. The strong preference for improving parent-child relationships aligns with core parenting program goals, while consideration of other outcomes reflects practitioners’ holistic approach to implementation. These findings can inform the design of interventions and implementation strategies that balance effectiveness with real-world constraints, potentially enhancing parenting programs’ adoption, sustainability, and impact.

Contributions to the literature

- This study pioneers the application of discrete choice experiments in exploring fidelity-adaptation decisions.
- Quantifying practitioners’ outcome preferences provides empirical evidence of complex trade-offs in implementing evidence-based parenting programs.
- The study’s results provide actionable insights for program developers, policymakers, and implementation scientists on designing interventions that resonate with practitioners’ priorities while maintaining program effectiveness.
- This research bridges theoretical frameworks and real-world decision-making, advancing our understanding of how various factors influence intervention delivery in practice.

## Background

A crucial aspect of evidence-based program implementation is managing fidelity-adaptation decisions. Fidelity, the degree to which interventions are delivered as intended by their developers, is often seen as essential for achieving outcomes in practice that are similar to those in program evaluation trials [1–3]. However, the contexts in which interventions are implemented tend to differ from those where the interventions were developed, with various needs, restraints, and other complex concerns competing for prioritization [4, 5]. While maximizing fidelity may protect intervention outcomes and reduce unfair or potentially risky variation of services, deliberate changes or modifications to the intervention (i.e., adaptations) may improve other outcomes. For example, adapting interventions to be more inclusive toward minority populations can enhance satisfaction and engagement with the service [6]. There are also reviews showing that adaptations can make some programs even more effective, such as programs that are transported across national contexts tend to show better effects if they are adapted instead of simply adopted without adaptation [7, 8].

Recent research has shifted away from viewing adaptation and fidelity as opposing forces, recognizing instead that appropriate adaptations can often enhance fidelity and implementation outcomes. Scholars have highlighted how flexibility within fidelity allows interventions to remain effective while responding to contextual needs [9]. This perspective acknowledges that interventions must evolve within changing delivery systems, as maintaining rigid adherence without contextual consideration can lead to poor implementation outcomes or program abandonment. The key consideration has thus shifted from whether to adapt, to understanding which adaptations can be made while maintaining intervention effectiveness. This evolution in thinking emphasizes the importance of ensuring that adaptations preserve essential elements of interventions while enhancing fit with local contexts [4, 10, 11]. However, implementing and delivering interventions involve multiple parallel outcomes that can be synergistic, contradictory, or unrelated to fidelity. This complexity has sparked discussions about whether adaptation and fidelity are better understood as a multicomponent decision-task in which the value of interventions in specific contexts should be assessed by considering the impact across all relevant outcomes [12]. This multifaceted approach reflects the reality of decision-making in practice, where practitioners must weigh various factors simultaneously when considering how to best resolve fidelity–adaptation dilemmas. Although a full assessment of possible outcomes is unrealistic mainly due to resource constraints, exploring which outcomes are considered, which are favored, and which are avoided remains crucial.

While efforts have been made to clarify the range of outcomes relevant to implementation science [13], there is a lack of research on how preferences for outcomes might influence decision-makers selection between fidelity and adaptation, especially in situations involving conflicting outcomes and forced trade-offs. This study aims to contribute to the fidelity-adaptation literature by exploring practitioners’ stated preferences for outcomes relevant to fidelity-adaptation decisions. Specifically, the study investigates how outcome preferences influence practitioners’ decisions to modify group-based parenting interventions. These programs aim to enhance parenting skills, foster healthy child development, and mitigate behavioral problems in children [14]. In the Swedish context, parenting programs are used in a variety of contexts (e.g., schools, primary care, hospitals, social services) by providers in various professions (e.g., social workers, psychologists, teachers, family therapists) and across the whole Continuum of Care (e.g., universal, selective, indicated). This variability makes parenting programs especially suitable for exploring differences among outcome preferences.

By focusing on outcome preferences in fidelity-adaptation dilemmas, we seek to evaluate their influence on practitioners’ choices and what kind of trade-offs they are willing to make. We will address the following research question: What is the relative importance of outcome preferences for influencing practitioners’ choices across adaptation scenarios typical for parenting programs?

## Methods

We employed a Discrete Choice Experiment (DCE) designed for practitioners delivering evidence-based parenting programs. A DCE is a quantitative method used in fields such as health economics [15], healthcare [16], marketing [17], and transportation [18] to elicit preferences by asking respondents to choose between sets of alternatives that vary across attributes. Attributes are the characteristics of the situation assumed to be important for choices, and values or variations within attributes are called levels. In the present study, we used outcomes relevant to resolving fidelity-adaptation dilemmas in the context of parenting programs (e.g., Relationship Quality, Satisfaction, Reach) as attributes. The procedure for selecting which outcome to choose as attributes is explained below.The underlying principle of deriving a categorical judgment from information-bearing cues is the cornerstone of cognitive research in psychology’s broader judgment- and decision-making research [19]. This methodology has also been beneficial for evaluating and comparing preferences involving trade-offs between conflicting attributes [20], making it suitable for the present study. 

### Participants and recruitment

Professionals from various parts of Sweden’s welfare system, such as municipality-employed social service and primary care providers, trained and experienced in delivering evidence-based parenting programs, were targeted for inclusion. Recruitment was made through two separate channels. First, program developers and agencies responsible for the training and quality assurance of parenting programs in Sweden, as listed on the Swedish Family Law and Parental Support Authority’s web page, were asked to distribute the survey to their trained personnel. Second, municipalities were directly contacted to distribute the survey locally to members of their staff trained in parenting programs. This dual-channel approach aimed to maximize participation from relevant professionals across different regions and settings.

Following recommendations from de Bekker-Grob et al.’s practical guide on sample size requirements for discrete-choice experiments in healthcare [21], the target sample size was set at a minimum of 120 participants during the initial planning of the study [22]. Two hundred nine group leaders agreed to participate in the study (Table 1). The average age was 47.7 (*SD *= 10.1, range=22–67), with most identifying as female (*n *= 187) compared to male (*n *= 22). A significant proportion (93 %) had either a bachelor’s, master’s, or higher degree (master’s and higher were not separated in the survey). The most common professions represented in the sample were Social Workers (*n *= 108, 52 %) and Pre/primary-school teachers (*n*=33, 16 %), with various other professions (e.g., nurses, physiotherapists, psychologists, counselors, health promotors, recreational leaders) being less frequent. Participants were trained in a single program (*n *= 115, 55%) or multiple programs (*n *= 94, 45%), with the most common programs represented being All Children in Focus (*n *= 128, 68 %) [23], Comet (*n *= 64, 30.5 %) [24], and Parenting in Sweden (22.9 %), indicating a broad representation of the most prevalent evidence-based parenting programs within the Swedish context. International programs represented were COS-P (14.3 %), COPE (10 %), Connect (7.1 %), and ICDP (5.2 %), and the remaining participants selected “Other” in the survey and noted various locally developed programs. One participant was excluded because of not providing any information regarding training in any evidence-based parenting program.

**Table 1:** Demographic information of participants agreeing to participate in the study

Variable	Description	Frequency (n)	Percentage (%)
Education	Master’s Degree or Higher	121	57.9 %
	Bachelor’s Degree	72	34.4 %
	High School Diploma	9	4,3 %
	Other Adult Education^1^	7	3 %
Experience	Range: 0.5–25 years		
	Mean ± SD: 6.6 ± 5.23		
Parenting programs	All Children in Focus	128	61 %
	Comet	64	30.5 %
	Parenting in Sweden	48	22.9 %
	Circle of Security Parenting (COS-P)	30	14.3 %
	COPE	21	10 %
	Connect	15	7.1 %
	International Child Development Programme (ICDP)	11	5.2 %
	Locally developed programs^2^	46	21.9 %

^1^Adult education programs unique to the Nordic countries encompassing: 1) Community Education Centers, offering non-degree lifelong learning and personal development courses; 2) Vocational Training, providing specific skills for trades or professions, often leading to certifications; and 3) Vocational Universities, focusing on higher education with practical skills for specific vocational careers. Each pathway supports diverse adult education and professional development needs. ^2^A variety of locally developed programs, some of which share similarities with established programs (e.g., COPE, Connect). Other programs meet specific needs of the local population or promote a topic of concern in the local community (e.g., managing divorce and children as victims of crime)

### DCE design

Designing a DCE involves several key considerations to ensure that it effectively captures the preferences of the target population. Our process was guided by the ISPOR Good Research Practices for Conjoint Analysis [25]. Following their recommendation, special care was taken to identify and select attributes and levels relevant to the study perspective. The procedures used are described in detail below.

#### Attribute identification and selection

**Fig. 1:**
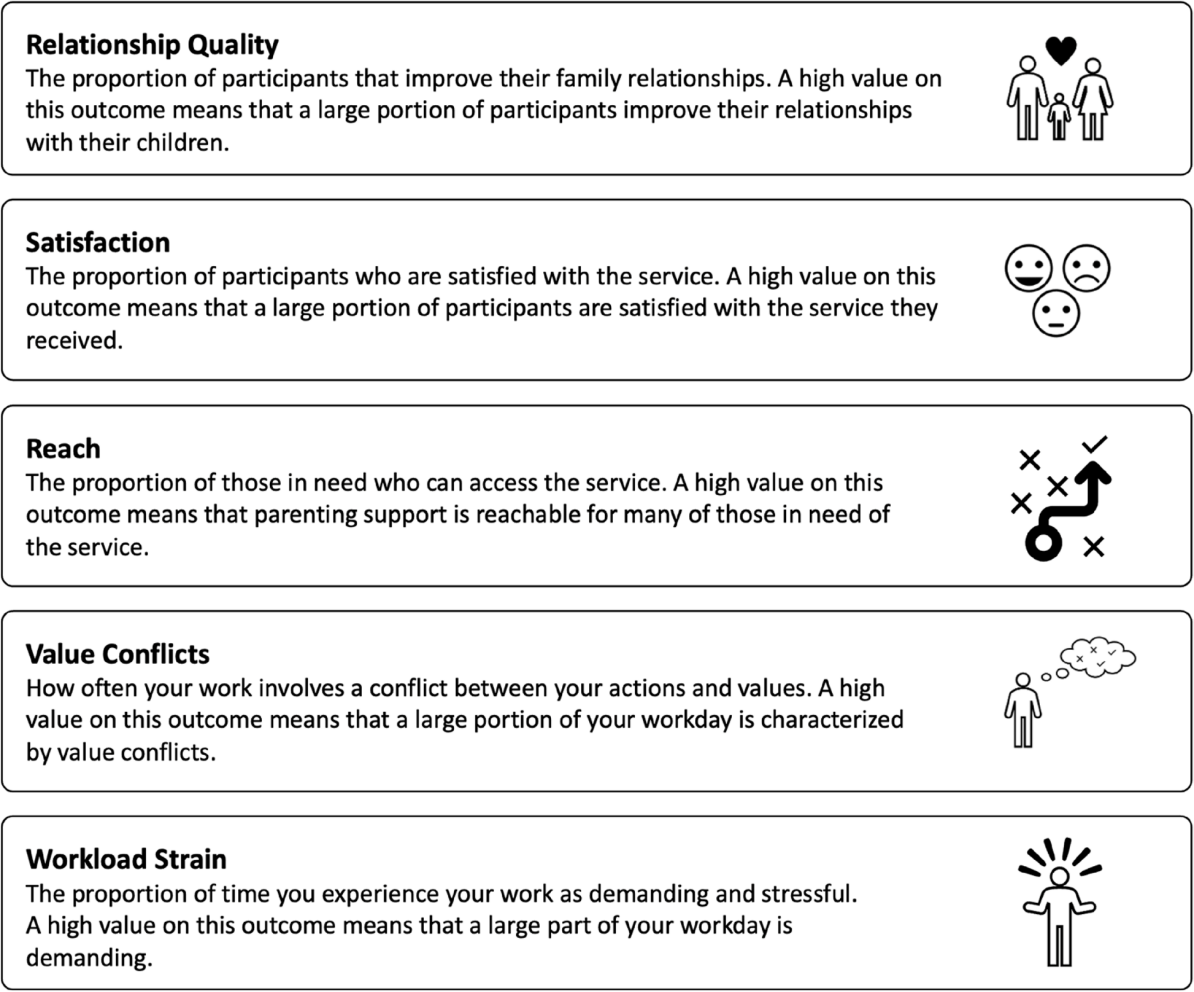
Definition of attributes as presented to participants

To assess outcome preference, the first step is to create the set of attributes on which the respondents base their decisions. To identify these outcomes, the research team conducted an exploratory study to generate important outcomes in parenting programs. Candidate attributes were listed and further elaborated based on outcome measures typically used in evaluation trials of parenting programs [26–28], as well as outcomes typically considered during implementation projects [13]. The list of attributes was then further expanded by adding outcomes mentioned in training manuals of some of the most widely disseminated parenting programs in Sweden [24, 29–32]. Next, all candidate attributes were processed during a two-day workshop in which the research group worked through and made the final selection for inclusion into the study. In this process, the team worked collaboratively to identify overlap among attributes, assess their relevance to adaptation decisions, and reduce the number of attributes to avoid overloading participants while maintaining strong statistical signal properties. In cases where overlap was identified, we strived toward identifying the more general and overarching outcome, hoping to cover as much as possible. The final list of selected attributes is summarized in Figure 1, while our rationale for their relevance to the study’s objective is described below.

##### Parent–child relationship quality

Intervention outcomes are central to fidelity-adaptation decisions [5, 12] and were thus deemed critical to include. However, outcome measures used to evaluate parenting programs cover several areas, such as various aspects of children’s and parents’ well-being and the quality of parent-child relationships [26]. We selected parent–child *relationship quality* because it is widely regarded as a crucial proximal outcome and mediating factor for program outcomes [23, 24, 29, 30, 32].

##### Parent satisfaction

High levels of parent *satisfaction* have been associated with continued engagement with the intervention [33], especially among ethnic minority groups [34]. Furthermore, practitioners sometimes view satisfaction as a critical indicator of a program’s success [35, 36]. Conceptually, we reasoned that satisfaction could be a unique outcome that may introduce a goal conflict with other outcomes. For these reasons, we decided to include satisfaction as an attribute.

##### Reach

*Reach* refers to the degree to which a program, practice, or intervention is delivered to the intended target population [37]. Although other concepts (e.g., adoption, penetration, and acceptability) offer more nuanced insights into the mechanisms through which reach is achieved and maintained, we selected reach for inclusion in the study because it represents an overarching view of the breadth and depth of an intervention’s impact within a community. Furthermore, several implementation frameworks incorporate reach as a consideration in fidelity-adaptation decisions [11, 38, 39], making it plausible to assume that practitioners might consider this factor when making fidelity-adaptation decisions.

##### Value conflict

*Value conflicts* occur when an intervention’s conceptual models, goals, or ethical frameworks diverge from the practitioners’ personally held beliefs about the origins of maladaptive behaviors and the strategies for addressing them. This phenomenon is generally referred to as *philosophical fit* [40], yet we decided to call it value conflict in this study to facilitate a clear presentation to participants. Value conflicts not only lead to emotional discomfort [41], but are also recognized as a potentially crucial factor for resolving fidelity-adaptation dilemmas [11, 12, 42].

##### Workload strain

Grounded in the Job Demands-Resources (JD-R) model, *workload strain* refers to job demands that can, over time, deplete practitioners’ physical and psychological resources, leading to burnout and reduced job satisfaction [43]. We reasoned that because of its detrimental effect on an individual’s health and well-being, the decision to adapt interventions may be significantly influenced by the need to manage workload strain effectively, making it suitable for inclusion as an attribute in the present study.

##### Level selection

The levels for each attribute in our Discrete Choice Experiment (DCE) were determined through a pilot study employing a think-aloud protocol [44] with ten participants from various parenting programs and professions. Participants were first briefed on each attribute and given the opportunity to ask clarifying questions. They were then asked to identify an acceptable level for each attribute and the range of variation they could tolerate if other attributes were improved. This process aimed to establish both a baseline acceptability and the boundaries of acceptable change for each attribute.

**Fig. 2:**
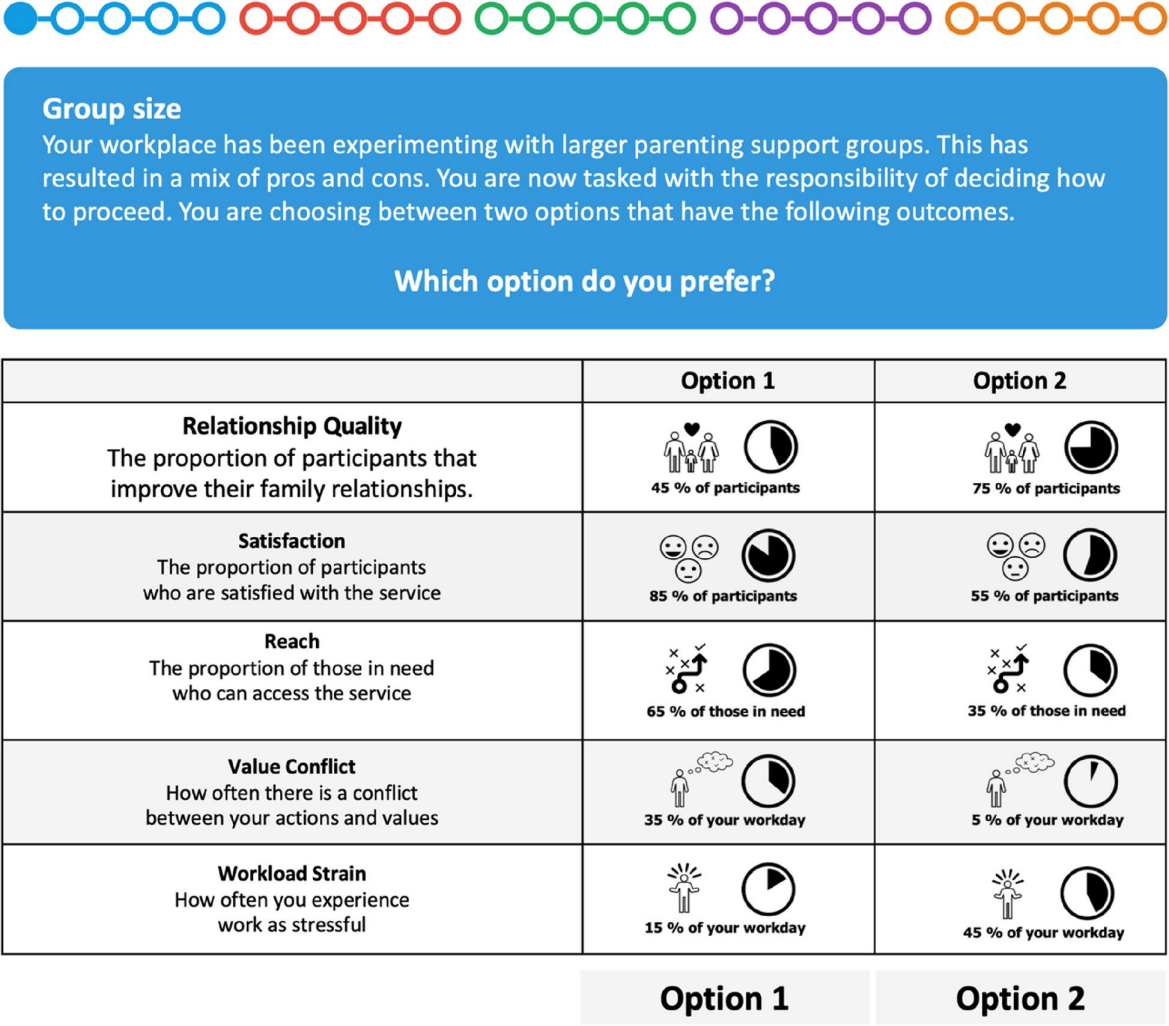
Example of a choice set included in trials. At the top, colored dots show progress, with colors indicating the scenarios framing the question as a fidelity-adaptation decision. The first column provides descriptions of attributes, while the second and third columns show verbal descriptions and illustrations for attribute levels. Participants were asked to select their preferred configuration of outcomes (the specific combination of levels across attributes) by selecting one of the buttons at the bottom of the screen

Analysis of the pilot data revealed an average tolerance of a 15 percentage point deviation from the mean value for each attribute. Consequently, the levels for each attribute were set at 15 percentage points above and below the mean values indicated by the pilot data, resulting in the levels included in the example choice set below (Figure 2). Levels were standardized as percentages to facilitate direct comparison across attributes.

##### Design and presentation of the survey

The survey was designed and distributed using Qualtrics^XM^. Initially, participants were introduced to the research project through an introductory section outlining the study’s purpose. After presenting the research objectives, participants were required to provide informed consent to continue with the survey. Ethical approval for the study involving human subjects was obtained from the Swedish Ethical Review Authority (reference no. 2021–00832).

Following consent, the survey solicited demographic background information. The subsequent survey section introduced the discrete choice experiment (DCE). Before commencing the experiment, participants were briefed on the study’s attributes: *Relationship Quality*, *Satisfaction*, *Reach*, *Value* *Conflict*, and *Workload Strain*. An example of a question with choice sets of two configurations across the five attributes was presented (Figure 2). Each option was described verbally and supplemented with visual aids to enhance comprehension, asking participants to select their preferred outcome configuration by clicking either Option 1 or Option 2 under the respective columns.

**Fig. 3:**
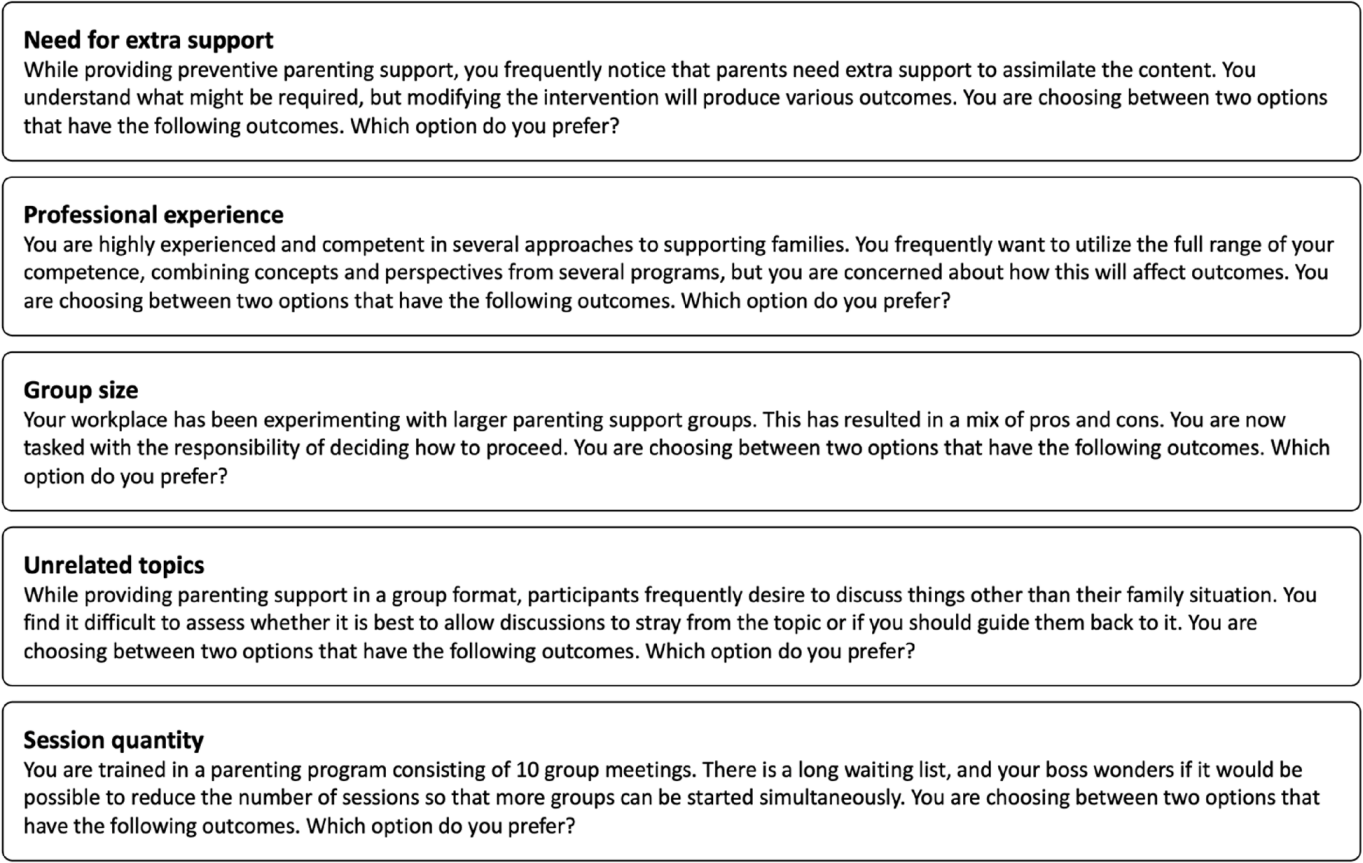
Description of the five scenarios preceding choice sets

We derived from our initial pilot testing that 25 trials would be a reasonable target given the relative familiarity of the topic [25, 45] and that the number of trials would yield informative posterior distributions for the regression analyses. The 25 trials were presented in five blocks with separate introductory scenario statements for each block (Figure 3). Each scenario was designed to present practitioners with choices that had implications for fidelity-adaptation concerns. However, to avoid introducing confounding variables, we could not explicitly state what changes would produce what outcome. For example, in the scenario describing group size modifications, it is implied that one option represents using bigger groups, while the other represents the standard group size. A progress bar was displayed at the top throughout the DCE, with colors alerting participants that a new scenario was presented. The scenarios were derived from one of our previous qualitative studies [36]. They served two purposes: (1) to prompt consideration of fidelity-adaptation decisions and (2) to contextualize the survey in real-world issues typical for parenting programs. To avoid order effects, the position of attributes, levels, colors, and scenarios were randomized across participants.

### Analytic approach

The data were analyzed using a Bayesian Hierarchical Logistic Regression model, implemented in the ‘brms’ package (Bayesian Regression Models using ‘Stan’; Version 2.21.0) within the R environment (Version 4.3.3), and facilitated by RStudio (Version 2023.12.1+402; RStudio, PBC). The five attributes consisted of predictor variables of participant choices. To account for individual differences, trial-specific effects, and contextual effects, we included Participant, Trial, and Scenario as random effects. The analysis was employed with four chains, each running for 4000 iterations with a warm-up period of 1000 iterations, resulting in 12,000 post-warmup draws (3000 post-warmup draws per chain).

To ensure that the direction of the model’s estimates directly reflected the preference impact, we used calculated differences for the levels presented in each trial by subtracting the value of levels presented in Option 1 from those presented in Option 2. For example, if Option 1 for the attribute *Satisfaction* was 85 % in a specific trial, and Option 2 was 55 %, the difference would be .30 (30 percentage point difference). Comparisons across attributes were made possible because the calculated differences across attributes could be either positive (.30), negative (-.30), or zero (0), depending on the position of levels in the choice set.

While the logistic regression model inherently estimates changes in log odds associated with predictor variables, we converted and reported these to odds ratios to facilitate interpreting and describing the results.

## Results

The Bayesian analysis of practitioners’ prioritization of outcomes when considering modifications to group-based parenting interventions revealed reliable effects across all attributes (Table 2), suggesting that all outcomes, to varying degrees, are relevant to how fidelity-adaptation dilemmas are resolved. Most influential was *Relationship Quality*, with an estimated log-odds of 4.56 (95% CI [4.16, 4.91]), suggesting a strong preference among practitioners for outcomes that enhance the quality of parent-child relationships. Participants were about 94 times more likely to select options containing 75 % than 45 % of parent-child improvements.

**Table 2:** Summary of estimated effects (Bayesian hierarchical logistic regression)

	Odds ratio	Log odds	Est. error	l-95% CI	u-95% CI
Intercept	0.98	−0.02	0.07	−0.17	0.13
Relationship Quality	93.99	4.56	0.19	4.16	4.91
Satisfaction	11.59	2.45	0.17	2.11	2.78
Value Conflict	0.09	−2.40	0.17	−2.75	−2.06
Workload Strain	0.12	−2.10	0.18	−2.44	−1.76
Reach	7.10	1.96	0.17	1.61	2.30

Less impactful was *Satisfaction*, with a log odds of 2.45 (95% CI [2.11, 2.78]), similar to the effect of *Value Conflict*, with a log odds of −2.4 (95% CI [−2.75, −2.06]). Thus, participants were about 11 times more likely to maximize *Satisfaction* by selecting options containing 85 % instead of 55 %, and to minimize *Value Conflict* by selecting 5 % instead of 35 %.

*Workload Strain* and *Reach* were slightly less important for resolving fidelity-adaptation decisions, with log odds of −2.1 (95% CI [−2.44, −1.76]) and 1.96 (95% CI [1.61, 2.30]), respectively. Participants were about eight times more likely to minimize workload strain by selecting options containing 15 % instead of 45 %. Similarly, participants were about seven times more likely to select options to reach 65 % instead of 35 % of those in need.

The model’s convergence was confirmed with Rhat values at 1.00 for all parameters, suggesting that the posterior distributions were well estimated. The effective sample sizes (Bulk_ESS and Tail_ESS) for each parameter were satisfactory, indicating reliable estimates and sufficient mixing of the Markov chains. Participant-specific effects were estimated at 0.12 (95% CI: 0.01 to 0.27), suggesting a minimal impact on choice compared to attributes. Trial-specific effects showed a standard deviation of the intercept at 0.06 (95% CI: 0.00 to 0.16), suggesting minor choice differences across trials and, thus, minimal or no ordering effect. Similarly, scenario effects were estimated at 0.12 (95% CI: 0.00 to 0.40), indicating similarities in choice across scenarios, thus suggesting that each scenario’s context or specific conditions did not markedly affect the participants’ choices. These effects demonstrated convergence (Rhat = 1.00), indicating reliable estimates from the model.

## Discussion

A discrete choice experiment was utilized to explore outcome preferences in fidelity-adaptation scenarios characteristic of parenting program delivery. Five outcomes were studied (*Relationship Quality*, *Satisfaction*, *Workload Strain*, *Value Conflict*, and *Reach*), revealing a substantial effect for *Relationship Quality*. Relative to other outcomes, options that improve parent-child relationships were greatly favored, although all outcomes influenced choice. This multi-component effect highlights the complexity involved in navigating fidelity-adaptation dilemmas, and it is the first study of its kind that assesses the influence of these factors experimentally in a manner that mimics the forced nature of real-world decisions where trade-offs are necessary.

The prominence of *Relationship Quality *as a determining factor for choice aligns with the importance placed on enhancing parent-child interactions as central to the success of parenting interventions [31, 32, 46, 47]. It is, after all, the primary target of parenting programs, and reviews suggest that interventions targeting this aspect of family dynamics are crucial for their success [26, 28, 48]. Thus, in the Swedish context, program developers and practitioners seem aligned in prioritization of intervention effects, which is not always assumed to be the case [12, 13, 40]. Although less influential, the pursuit of *Satisfaction* and *Reach*, and avoidance of increased *Value Conflicts *and *Workload Strain*, still showed an effect on choice, indicating that practitioners, in addition to intervention effects, also value dissemination and acceptability of interventions and their own well-being. This finding aligns with the dissemination and implementation (D&I) models that advocate for scaling interventions while ensuring that they are adapted to meet the needs of diverse populations [49].

The willingness to consider multiple outcomes, with a clear prioritization of intervention outcomes, suggest that practitioners are inclined to preserve essential elements of programs when considering adaptations, providing a foundation for guided adaptation processes. Implementation support could build on this tendency by helping practitioners to identify which program elements are essential for maintaining effectiveness and which can be modified to enhance local fit [50]. Training and implementation strategies could focus on developing practitioners’ capacity to make informed decisions about adaptations, rather than emphasizing strict adherence to original protocols. This approach would align with contemporary implementation science frameworks that recognize the value of thoughtful, contextually-appropriate modifications [10, 51, 52].

The negative impacts of *Value Conflict *and *Workload Strain* on decision-making highlight how practitioner-level barriers can influence implementation choices. When levels are increased, *Value Conflict *and *Workload Strain* could compete with other favored outcomes, potentially posing significant barriers to intervention fidelity [53]. This exemplifies the practical constraints that can limit the flexibility of implementing evidence-based practices in real-world settings and points to the need to carefully consider the practitioners’ working conditions and belief systems when introducing new programs or adaptations. This dual focus acknowledges that sustainable implementation requires both maintaining intervention effectiveness and creating supportive conditions for delivery. Identifying and minimizing these factors may enhance interventions’ adoption and sustainability while protecting practitioners’ well-being. The disproportionally strong effect of intervention outcomes on practitioners’ choice might allow implementers of parenting programs to spend time addressing potential barriers since group leaders’ tendency to optimize parent-child relationship quality does not need much encouragement.

### Methodological considerations

Our DCE design followed established guidelines, with rigorous procedures for identifying relevant scenarios, attributes, and levels. Thus, effects are reliably estimated for the target population (providers of parenting programs) and their specific context (the Swedish health and welfare system). However, the relevance of studied outcomes (Relationship Quality, Satisfaction, Value Conflict, Workload Strain, and Reach) will likely vary across interventions, settings, and cultures. It is worth pointing out that Sweden today represents a multicultural society with diverse ethnic backgrounds and cultural perspectives, thereby enhancing the relevance of findings to other cultural settings. However, the relative prioritization of these outcomes may still vary across different cultural or geographical contexts. Thus, while our findings offer valuable, broadly applicable insights, future research could explore how these priorities might shift across other settings, cultures, and interventions.

The disproportionate influence of Relationship Quality in our findings must be considered within the context of our attribute selection. As the only intervention outcome among our measured attributes, its prominence may reflect practitioners' broader prioritization of intervention outcomes over implementation concerns, rather than a specific preference for relationship quality improvements. Future research could benefit from including multiple intervention outcomes alongside implementation outcomes. This would enable a more comprehensive understanding of how practitioners prioritize different aspects of both intervention effectiveness and implementation success. For instance, studies could examine how practitioners weigh different intervention outcomes (e.g., behavioral change, skill acquisition, relationship quality) against various implementation outcomes (e.g., feasibility, sustainability, cost-effectiveness). This could also involve examining how different combinations of implementation and intervention outcomes influence adaptation decisions.

Expanding future research to explore a more comprehensive array of attributes is essential for improving the model’s robustness and relevance across diverse implementation environments. However, decisions to incorporate more attributes into Discrete Choice Experiments (DCEs) must be weighed against the risk of cognitive overload, which can compromise the reliability of participant responses due to the complexity of decision scenarios [25].

Our data suggest that a certain level of one of the attributes with a positive effect could be canceled out by one of the negative attributes at the level of the corresponding probability. This, however, assumes a linear impact on choice across attribute levels. In reality, the influence of attributes may not scale directly with their levels. Some attributes might, for example, display threshold effects that mark a shift in influence on choice at a certain level. It is also possible that attributes could interact and produce effects that are not predictable by considering them independently. We decided not to pursue these questions, partly due to our limited sample size but also because of our study’s explorative nature.

To summarize, several limitations should be considered. First, the discrete choice experiment explored only a limited set of attributes, potentially overlooking other factors that might influence practitioners’ decisions in fidelity-adaptation scenarios. Second, while efforts were made to ensure ecological validity, the experimental nature of the choice tasks may only partially capture the complexity of real-world decision-making. Third, the study’s focus on the Swedish context may limit its generalizability to other cultural or healthcare systems. Additionally, the assumption of linear effects across attribute levels may oversimplify the nuanced dynamics of practitioner preferences. Future research could address these limitations by iterating on attribute selection, incorporating more diverse cultural contexts, and exploring potential non-linear effects or interactions between attributes.

### Implications

The present study is the first to employ a Discrete Choice Experiment to explore the role of outcome preferences in resolving fidelity-adaptation dilemmas. This method revealed that practitioners approach adaptation decisions with a clear hierarchy of priorities, suggesting that the fidelity-adaptation tension might be better understood as a question of maintaining essential outcomes while allowing flexibility in delivery methods. Rather than viewing fidelity and adaptation as competing forces, these findings support an approach where adaptation decisions are guided by their impact on critical outcomes. From this perspective, debates on how to best resolve fidelity–adaptation dilemmas can be construed as differing views on what outcomes to prioritize and how best to achieve them. As implementation science continues embracing a multilevel and multifactorial analysis of outcomes [4, 5, 12, 54], there is a considerable need for advancing methods to grasp this complexity. Discrete Choice Experiments (DCE) offer a coherent approach to exploring some of these real-world dynamic processes while retaining more experimental control than what is often feasible outside the laboratory.

When implementing and working on continuous improvements of interventions, it may be worthwhile for organizations to systematically consider which outcomes are locally important and to what extent they influence decisions about intervention delivery. This could involve workshops that support practitioners in identifying goals and finding common ground on how to achieve them [50]. Such activities could enhance alignment between organizational objectives and practitioners’ preferences, potentially leading to more effective and sustainable implementation efforts.

Our findings support the view of front-line practitioners as active and responsible in managing the complexities of their applied decision-making. Knowledge of how outcome preferences influence decision-making in real-world scenarios is valuable not only to those working in these settings but also to all those providing support in doing so. The comprehensive understanding of practitioners’ outcome preferences that this study provides can inform program developers to tailor interventions to meet the needs of both practitioners and recipients.

Future research in this area could benefit from assessing the extent to which practitioners value the theoretical mechanisms through which any given intervention operates. For instance, studies of adaptation decisions in cognitive behavioral therapy (CBT) could examine how practitioners prioritize changing cognitions relative to other outcomes. This approach could provide valuable insights into the alignment between theoretical foundations and practical implementation across various intervention types.

## Conclusions

This study provides crucial insights into the outcome preferences influencing practitioners’ decisions in fidelity-adaptation dilemmas. It reveals that while relationship quality is the primary driver of decisions, practitioners consider factors such as satisfaction, value conflicts, program reach, and workload. The study uncovers potential tensions between clinical effectiveness and practical considerations, demonstrating the complex nature of fidelity-adaptation decisions. We offer a novel approach to understanding these multifaceted choices by employing Discrete Choice Experiments. These findings underscore the need for a nuanced approach to implementation that balances intervention effectiveness with practical realities. Program developers and policymakers can use these insights to design interventions that maintain effectiveness while aligning with practitioners’ needs, potentially enhancing parenting programs’ adoption, sustainability, and overall impact.

## Data Availability

The datasets used will be available from the corresponding author upon reasonable request.

## References

[1] Durlak JA, DuPre EP. Implementation matters: a review of research on the influence of implementation on program outcomes and the factors affecting implementation. Am J Community Psychol. 2008;41(3–4):327–50. 18322790 10.1007/s10464-008-9165-0

[2] Hansen WB, Graham JW, Wolkenstein BH, Rohrbach LA. Program integrity as a moderator of prevention program effectiveness: Results for fifth-grade students in the adolescent alcohol prevention trial. J Stud Alcohol. 1991;52(6):568–79. 1758184 10.15288/jsa.1991.52.568

[3] Dusenbury L, Brannigan R, Falco M, Hansen WB. A review of research on fidelity of implementation: implications for drug abuse prevention in school settings. Health Educ Res. 2003;18:237–56. 12729182 10.1093/her/18.2.237

[4] Chambers DA, Glasgow RE, Stange KC. The dynamic sustainability framework: addressing the paradox of sustainment amid ongoing change. Implement Sci. 2013;8(1):1–11. 24088228 10.1186/1748-5908-8-117 PMC3852739

[5] Kirk MA, Moore JE, Wiltsey Stirman S, Birken SA. Towards a comprehensive model for understanding adaptations’ impact: the model for adaptation design and impact (MADI). Implement Sci. 2020;15(1):1–15. 32690104 10.1186/s13012-020-01021-y PMC7370455

[6] Kumpfer K, Magalhães C, Xie J. Cultural adaptation and implementation of family evidence-based interventions with diverse populations. Prev Sci. 2017;18(6):649–59. 27757773 10.1007/s11121-016-0719-3

[7] Sundell K, Beelmann A, Hasson H, von Thiele SU. Novel programs, international adoptions, or contextual adaptations? Meta-analytical results from German and Swedish intervention research. J Clin Child Adolesc Psychol. 2016;45(6):784–96. 25864716 10.1080/15374416.2015.1020540

[8] Olsson TM, von Thiele Schwarz U, Hasson H, Vira EG, Sundell K. Adapted, Adopted, and Novel Interventions: A Whole-Population Meta-Analytic Replication of Intervention Effects. Res Soc Work Pract. 2023;34(8):860-72.

[9] Kendall PC, Frank HE. Implementing evidence-based treatment protocols: flexibility within fidelity. Clin Psychol (New York). 2018;25(4):40. 10.1111/cpsp.12271 PMC6329472 30643355

[10] Chambers DA, Norton WE. The adaptome: advancing the science of intervention adaptation. Am J Prev Med. 2016;51(4 Suppl 2):S124–31. 27371105 10.1016/j.amepre.2016.05.011 PMC5030159

[11] Wiltsey Stirman S, Baumann AA, Miller CJ. The FRAME: an expanded framework for reporting adaptations and modifications to evidence-based interventions. Implement Sci. 2019;14(58):1–10. 31171014 10.1186/s13012-019-0898-y PMC6554895

[12] von Thiele SU, Aarons GA, Hasson H. The value equation: three complementary propositions for reconciling fidelity and adaptation in evidence-based practice implementation. BMC Health Serv Res. 2019;19(1):1–10. 31752846 10.1186/s12913-019-4668-y PMC6873662

[13] Proctor E, Silmere H, Raghavan R, Hovmand P, Aarons G, Bunger A, et al. Outcomes for implementation research: conceptual distinctions, measurement challenges, and research agenda. Adm Policy Ment Health. 2011;38(2):65–76. 20957426 10.1007/s10488-010-0319-7 PMC3068522

[14] National Research Council and Institute of Medicine. Preventing mental, emotional, and behavioral disorders among young people: progress and possibilities. Washington, DC: The National Academies Press; 2009. Report No.: 978-0-309-12674-8. 20662125

[15] Clark MD, Determann D, Petrou S, Moro D, de Bekker-Grob EW. Discrete choice experiments in health economics: a review of the literature. Pharmacoeconomics. 2014;32(9):883–902. 25005924 10.1007/s40273-014-0170-x

[16] Wang Y, Wang Z, Wang Z, Li X, Pang X, Wang S. Application of discrete choice experiment in health care: a bibliometric analysis. Front Public Health. 2021;9: 673698. 34150710 10.3389/fpubh.2021.673698 PMC8212992

[17] Louviere JJ, Hensher DA, Swait JD. Stated choice methods : analysis and applications. Cambridge: Cambridge University Press; 2000. 10.1017/CBO9780511753831.

[18] Train K. Discrete choice methods with simulation. 2nd ed. Cambridge: Cambridge University Press; 2009.

[19] Newell BR, Lagnado DA, Shanks DR. Straight choices : the psychology of decision making. Abingdon, Oxon: Routledge; 2022. Available from: https://search.ebscohost.com/login.aspx?direct=trueandscope=siteanddb=nlebkanddb=nlabkandAN=3269004.

[20] Cleland J, Porteous T, Skatun D. What can discrete choice experiments do for you? Med Educ. 2018;52(11):1113–24. 30259546 10.1111/medu.13657

[21] de Bekker-Grob EW, Donkers B, Jonker MF, Stolk EA. Sample size requirements for discrete-choice experiments in healthcare: a practical guide. Patient. 2015;8:373–84. 25726010 10.1007/s40271-015-0118-z PMC4575371

[22] von Thiele Schwarz U, Lyon AR, Pettersson K, Giannotta F, Liedgren P, Hasson H. Understanding the value of adhering to or adapting evidence-based interventions: a study protocol of a discrete choice experiment. Implement Sci Commun. 2021;2(1):88. 34380575 10.1186/s43058-021-00187-w PMC8356451

[23] Ulfsdotter M, Enebrink P, Lindberg L. Effectiveness of a universal health-promoting parenting program: a randomized waitlistcontrolled trial of all children in focus. BMC Public Health. 2014;14(1):1–11. 25326710 10.1186/1471-2458-14-1083 PMC4210619

[24] Kling A, Forster M, Sundell K, Melin L. A randomized controlled effectiveness trial of parent management training with varying degrees of therapist support. Behav Ther. 2010;41(4):530–42. 21035616 10.1016/j.beth.2010.02.004

[25] Bridges JF, Hauber AB, Marshall D, Lloyd A, Prosser LA, Regier DA, et al. Conjoint analysis applications in health–a checklist: a report of the ISPOR good research practices for conjoint analysis task force. Value Health. 2011;14(4):403–13. 21669364 10.1016/j.jval.2010.11.013

[26] Barlow J, Coren E. The effectiveness of parenting programs. Res Soc Work Pract. 2018;28(1):99–102.

[27] Leijten P, Gardner F, Melendez-Torres GJ, van Aar J, Hutchings J, Schulz S, et al. Meta-analyses: key parenting program components for disruptive child behavior. J Am Acad Child Adolesc Psychiatry. 2019;58(2):180–90. 30738545 10.1016/j.jaac.2018.07.900

[28] Leijten P, Melendez-Torres GJ, Gardner F. Research review: the most effective parenting program content for disruptive child behavior – a network meta-analysis. J Child Psychol Psychiatry. 2021;63(2):132–42. 34240409 10.1111/jcpp.13483

[29] Moretti M, Holland R, Moore K, McKay S. An attachment-based parenting program for caregivers of severely conduct disordered adolescents: preliminary findings. J child youth care work. 2004;19(February):170–9.

[30] Cunningham C. Large group, community based, family-centered parent training. In: Barkley RA, Murphy KR, editors. Attention deficit hyperactivity disorder: a clinical workbook. New York: Guilford Press; 2005. p. 480–98.

[31] Enebrink P, Danneman M, Benvestito Mattsson V, Ulfsdotter M, Jalling C, Lindberg L. ABC for parents: pilot study of a universal 4-session program shows increased parenting skills, self-efficacy and child well-being. J Child Fam Stud. 2014;24(7):1917–31.

[32] Sanders MR, Triple P. – Positive parenting program: a population approach to promoting competent parenting. AeJAMH. 2014;2(3):127–43.

[33] Nock MK, Phil M, Kazdin AE. Parent expectancies for child therapy: assessment and relation to participation in treatment. J Child Fam Stud. 2001;10(2):155–80.

[34] Coatsworth JD, Duncan LG, Pantin H, Szapocznik J. Retaining ethnic minority parents in a preventive intervention: the quality of group process. J Prim Prev. 2006;27(4):367–89. 16802072 10.1007/s10935-006-0043-y

[35] Finan SJ, Warren N, Priest N, Mak JS, Yap MBH. Parent non-engagement in preventive parenting programs for adolescent mental health: stakeholder views. J Child Fam Stud. 2019;29(3):711–24.

[36] Pettersson K, Liedgren P, Giannotta F, von Thiele Schwarz U. Eleven reasons for adaptation of Swedish parenting programs. Front Health Serv. 2022;2:923504. 36925861 10.3389/frhs.2022.923504 PMC10012651

[37] Glasgow RE, Vogt TM, Boles SM. Evaluating the public health impact of health promotion interventions: the RE-AIM framework. Am J Public Health. 1999;89(9):1322–7. 10474547 10.2105/ajph.89.9.1322 PMC1508772

[38] Escoffery C, Lebow-Skelley E, Udelson H, Böing EA, Wood R, Fernandez ME, et al. A scoping study of frameworks for adapting public health evidence-based interventions. Transl Behav Med. 2019;9(1):1–10. 29346635 10.1093/tbm/ibx067 PMC6305563

[39] Glasgow RE, Battaglia C, McCreight M, Ayele R, Maw AM, Fort MP, et al. Use of the reach, effectiveness, adoption, implementation, and maintenance (RE-AIM) framework to guide iterative adaptations: applications, lessons learned, and future directions. Front Health Serv. 2022;2:2. 10.3389/frhs.2022.959565 PMC10012751 36925843

[40] Moore JE, Bumbarger BK, Cooper BR. Examining adaptations of evidence-based programs in natural contexts. J Prim Prev. 2013;34(3):147–61. 23605294 10.1007/s10935-013-0303-6

[41] Gray JS, Ozer DJ, Rosenthal R. Goal conflict and psychological well-being: a meta-analysis. J Res Pers. 2017;66:27–37.

[42] Moore G, Campbell M, Copeland L, Craig P, Movsisyan A, Hoddinott P, et al. Adapting interventions to new contexts—the ADAPT guidance. BMJ. 2021;374:n1679. 10.1136/bmj.n1679 PMC8329746 34344699

[43] Bakker AB, Demerouti E. Job demands-resources theory: taking stock and looking forward. J Occup Health Psychol. 2017;22(3):273–85. 27732008 10.1037/ocp0000056

[44] Noushad B, Van Gerven PWM, de Bruin ABH. Twelve tips for applying the think-aloud method to capture cognitive processes. Med Teach. 2024;46(7):892–7. 38071621 10.1080/0142159X.2023.2289847

[45] de Bekker-Grob EW, Ryan M, Gerard K. Discrete choice experiments in health economics: a review of the literature. Health Econ. 2012;21(2):145–72. 22223558 10.1002/hec.1697

[46] Patterson GR. Coercive family process. USA: Castalia publishing company; 1982.

[47] Moretti M, Pasalich D, O´Donnell K. Connect: An attachment‐based program for parents of teens. In: Steele H, Steele M, editors. Handbook of Attachment-Based Interventions. 1. First edit ed. New York: The Guilford Press; 2018. p. 375-400.

[48] Wyatt Kaminski J, Valle LA, Filene JH, Boyle CL. A meta-analytic review of components associated with parent training program effectiveness. J Abnorm Child Psychol. 2008;36(4):567–89. 18205039 10.1007/s10802-007-9201-9

[49] Greenhalgh T, Robert G, Macfarlane F, Bate P, Kyriakidou O. Diffusion of innovations in service organizations: systematic review and recommendations. Milbank Q. 2004;82(4):581–629. 15595944 10.1111/j.0887-378X.2004.00325.x PMC2690184

[50] Hasson H, Hedberg Rundgren E, Strehlenert H, Gärdegård A, Uvhagen H, Klinga C, et al. The adaptation and fidelity tool to support social service practitioners in balancing fidelity and adaptations: longitudinal, mixed-method evaluation study. Implementation Research and Practice. 2023;4:4. 10.1177/26334895231189198 PMC10392202 37790175

[51] Miller CJ, Wiltsey-Stirman S, Baumann AA. Iterative decision-making for evaluation of adaptations (IDEA): a decision tree for balancing adaptation, fidelity, and intervention impact. J Community Psychol. 2020;48(4):1163–77. 31970812 10.1002/jcop.22279 PMC7261620

[52] Brewer SK, Corbin CM, Baumann AA, Stirman SW, Jones JM, Pullmann MD, et al. Development of a method for Making Optimal Decisions for Intervention Flexibility during Implementation (MODIFI): a modified Delphi study. Implement Sci Commun. 2024;5(1):64. 10.1186/s43058-024-00592-x PMC11181660 38886834

[53] Grol R, Wensing M. What drives change? Barriers to and incentives for achieving evidence-based practice. Med J Aust. 2004;180(S6):S57-60. 15012583 10.5694/j.1326-5377.2004.tb05948.x

[54] Proctor EK, Bunger AC, Lengnick-Hall R, Gerke DR, Martin JK, Phillips RJ, et al. Ten years of implementation outcomes research: a scoping review. Implement Sci. 2023;18(1):31. 37491242 10.1186/s13012-023-01286-z PMC10367273

